# Antibiofilm effect of melittin alone and in combination with conventional antibiotics toward strong biofilm of MDR-MRSA and -*Pseudomonas aeruginosa*

**DOI:** 10.3389/fmicb.2023.1030401

**Published:** 2023-02-20

**Authors:** Rasoul Mirzaei, Hadi Esmaeili Gouvarchin Ghaleh, Reza Ranjbar

**Affiliations:** ^1^Department of Microbiology, School of Medicine, Hamadan University of Medical Sciences, Hamadan, Iran; ^2^Applied Virology Research Center, Baqiyatallah University of Medical Sciences, Tehran, Iran; ^3^Molecular Biology Research Center, Systems Biology and Poisonings Institute, Baqiyatallah University of Medical Sciences, Tehran, Iran

**Keywords:** MDR, MRSA, *Pseudomonas aeruginosa*, biofilm, antibiofilm peptide, melittin, synergism

## Abstract

**Introduction:**

Multidrug-resistant (MDR) pathogens are being recognized as a critical threat to human health if they can form biofilm and, in this sense, biofilm-forming MDR-methicillin resistant *Staphylococcus aureus* (MRSA) and -*Pseudomonas aeruginosa* strains are a worse concern. Hence, a growing body of documents has introduced antimicrobial peptides (AMPs) as a substitute candidate for conventional antimicrobial agents against drug-resistant and biofilm-associated infections. We evaluated melittin’s antibacterial and antibiofilm activity alone and/or in combination with gentamicin, ciprofloxacin, rifampin, and vancomycin on biofilm-forming MDR-*P. aeruginosa* and MDR-MRSA strains.

**Methods:**

Antibacterial tests [antibiogram, minimum inhibitory concentration (MIC), and minimum bactericidal concentration (MBC)], anti-biofilm tests [minimum biofilm inhibition concentration (MBIC), and minimum biofilm eradication concentration (MBEC)], as well as synergistic antibiofilm activity of melittin and antibiotics, were performed. Besides, the influence of melittin alone on the biofilm encoding genes and the cytotoxicity and hemolytic effects of melittin were examined.

**Results:**

MIC, MBC, MBIC, and MBEC indices for melittin were in the range of 0.625–5, 1.25–10, 2.5–20, and 10–40 μg/ml, respectively. The findings found that the combination of melittin AMP with antibiotics was synergistic and fractional biofilm inhibitory concentration index (FBICi) for most tested concentrations was <0.5, resulting in a significant reduction in melittin, gentamicin, ciprofloxacin, vancomycin, and rifampin concentrations by 2–256.4, 2–128, 2–16, 4–64 and 4–8 folds, respectively. This phenomenon reduced the toxicity of melittin, whereby its synergist concentration required for biofilm inhibition did not show cytotoxicity and hemolytic activity. Our findings found that melittin decreased the expression of *icaA* in *S. aureus* and *LasR* in *P. aeruginosa* genes from 0.1 to 4.11 fold for *icaA*, and 0.11 to 3.7 fold for *LasR*, respectively.

**Conclusion:**

Overall, the results obtained from our study show that melittin alone is effective against the strong biofilm of MDR pathogens and also offers sound synergistic effects with antibiotics without toxicity. Hence, combining melittin and antibiotics can be a potential candidate for further evaluation of *in vivo* infections by MDR pathogens.

## 1. Introduction

Multidrug-resistant (MDR) pathogens are widely noted as one of the most significant public health issues nowadays (van Duin and Paterson, 2020). MDR pathogens are typically related to nosocomial bacterial infections, and also MDR pathogens have become a common cause of bacterial community-acquired illnesses (van Duin and Paterson, 2020). Accordingly, a broader range of antibiotics and combination agents is advised for the empirical treatment of MDR infections when the occurrence of a given resistance pattern in bacterial infections surpasses a certain threshold, which can have negative outputs (van Duin and Paterson, 2016; Lertwattanachai et al., 2020; Bassetti and Garau, 2021). In this regard, antibiotic resistance among *Pseudomonas aeruginosa* strains is a growing concern, and some Extensively Drug-Resistant (XDR) *P. aeruginosa* have recently become issues of public health concern (Horcajada and Montero, 2019; Pang et al., 2019). On the other hand, *Staphylococcus aureus* infections are also deadly and difficult to treat over the current decades due to the rising frequency of antibiotic resistance (Foster, 2017). In this sense, MDR methicillin-resistant *S. aureus* (MRSA) is of great concern (Lee et al., 2018).

Of note, MDR pathogens can pose a substantial threat to patients due to biofilm formation (Sobisch et al., 2019). Aside from its resistance determinants, bacterial biofilm development is also a significant factor contributing to unsuccessful treatment attempts (Mahdiun et al., 2017; Mirzaei et al., 2020a). Biofilms are bacterial populations encased in an extracellular matrix that enhance bacterial adherence to various surfaces like the host cells (Mirzaei et al., 2020b, 2022b). This mode of growth is a crucial virulence factor in the development of some bacterial infections like wounds, device-associated infections, dental caries, and other chronic infections because of its resistance to antibiotics and protective barriers toward harsh environmental stressors and the immune system (Mirzaei and Ranjbar, 2022). In this aspect, antibiotic resistance in biofilm often happens during monotherapy; therefore, these antibiotics should usually be used as combination therapy with other antimicrobial agents (Howden et al., 2010; Bardbari et al., 2018). Antibiotic monotherapy is frequently ineffective in treating MDR infections; moreover, antibiotic-induced toxicity at higher concentrations in monotherapy necessitates careful monitoring of the patients (Mirzaei et al., 2022a). As a result, researchers discovered that combining antimicrobial peptides (AMPs) and antibiofilm peptides (ABPs) with antibiotics can be a viable therapy for treating MDR pathogens infections. Notably, AMPs, as part of the innate immunity of organisms, are a promising class of compounds that are currently receiving special attention as an emerging alternative to conventional antibacterial drugs against biofilm-producing MDR pathogens (Mahlapuu et al., 2016). In this way, melittin as a cationic AMP is well demonstrated against a wide range of bacterial pathogens alone, and this powerful AMP exhibit synergistic activity in combination with some antibiotics against different MDR species pathogens (Khozani et al., 2019; Zarghami et al., 2021b; Mirzaei et al., 2022a,b). As the main component of bee venom, melittin is one of the studied AMPs with strong antimicrobial activity, and its potential effects against viruses and cancer cells have also been found (Choi et al., 2015b; Askari et al., 2021). Hence, the current work was done to survey the effect of melittin AMP alone and/or in combination with gentamicin, ciprofloxacin, rifampin, and vancomycin on biofilm-forming MDR-MRSA and MDR-*P. aeruginosa*.

## 2. Methods

### 2.1. Antibiotics, media, and reagents

The present study provided the disks and powdered antibiotics from the MAST (Mast Diagnostics, United Kingdom) and Sigma-Aldrich (Taufkirchen, Germany). The following items were acquired from Merck (Merck, United States): Blood Agar, Mannitol Salt Agar (MSA), MacConkey agar, Cetrimide agar, Mueller Hinton Agar (MHA), DNA Agar, Mueller Hinton Broth (MHB), Trypticase Soy Broth (TSB), NaCl, glucose, and MgCl2. Fetal bovine serum (FBS), Dulbecco’s Modified Eagle’s Medium (DMEM), Fetal-Calf Serum (FCS), 3-(4, 5-dimethyl-2-thiazolyl)-2, 5-diphenyl-2 H-tetrazoliumbromide (MTT), Triton X-100, dimethyl sulfoxide (DMSO), agarose, ethanol, methanol, and crystal violet were provided from Sigma-Aldrich (Saint Louis, MO, United States). 96-well microplates, including flat-and round-bottom, were supplied by Jet Biofil (Guangzhou, China) and NEST Biotechnology (Wuxi, China), respectively.

### 2.2. Melittin synthesis order

The complete sequence of melittin peptide (GIGAVLKVLTTGLPALISWIKRKRQQ) blasted in NCBI with a purity of >96% was synthesized *via* the Solid-Phase method by DGpeptides (Hubei, China). In this regard, the corporation applied reversed-phase high-performance liquid chromatography to evaluate the purity of synthesized peptides. The company used liquid chromatography-mass spectrometry to verify accurate synthesis for mass spectrometry. Finally, the bicinchoninic acid test and reversed-phase high-performance liquid chromatography were used to confirm the peptide content and purity (Eisapoor et al., 2016).

### 2.3. Collection and confirmation of clinical isolates and standard strains

The Centers for Disease Control followed inclusion guidelines in this study (Horan et al., 2008). The isolates were obtained from individuals of varying ages and genders and were not duplicated; just one sample per patient was collected. In this regard, 30 *S. aureus* isolates were obtained from the wound (*n* = 8), blood (*n* = 11), urine (*n* = 6), as well as sputum (*n* = 5) and further characterized and confirmed using biochemical tests like colony morphology, gram-positive, clustered-shaped cocci, catalase, mannitol, DNase, and coagulase (Murray et al., 1995). In addition, 20 clinical *P. aeruginosa* were collected from respiratory tracts retrieved from sputum (*n* = 6), bronchoalveolar lavage (8), and endotracheal aspirates (*n* = 6) patients hospitalized in the intensive care unit (ICU) wards and then confirmed on selective media *via* conventional phenotypical tests such as colony morphology, oxidase, catalase, motility, citrate, indole synthesis, methyl red, and voges-proskauer. Finally, molecular confirmation of *S. aureus* and *P. aeruginosa* isolates was done by the polymerase chain reaction (PCR) *via* previously described primers (Atshan et al., 2012; Abdelraheem et al., 2020). Besides, *S. aureus* ATCC 25923, *S. aureus* ATCC 29213, and *P. aeruginosa* PAO1 were provided by the Pasteur Institute of Iran.

### 2.4. Screening for MRSA isolates

According to the Clinical and Laboratory Standards Institute (CLSI) 2020 recommendations, *S. aureus* isolates were phenotypically evaluated for resistance to methicillin using the cefoxitin (FOX; 30 μg) by Kirby-Bauer method (Wayne, 2010). In this regard, briefly, *S. aureus* colonies were cultivated in the MHB overnight at 37°C with 180 rpm shaking, and then the optical density (OD) of bacteria was set on 0.5 McFarland, followed by the suspension was swabbed onto the MHA plates, and then the FOX disk was placed and 24 h incubated at 37°C. Then, the diameter of the inhibition zone developed around the FOX disk was assessed. Then, MRSA isolates were genotypically confirmed using PCR *via* the *mecA* gene by the previously designed primer (Kelley et al., 2013). In brief, genomic DNA from colonies of isolates was extracted using the Purification kit (Roche, Germany) based on the manufacturer’s instruction, and PCR reactions were done in a 20 μl volume containing 1.5 μl MgCl2, 2.5 μl of PCR buffer (10X), 0.5 μl dNTP (10 mmol/l), 0.5 μl of each reverse and forward primers, 1 μl of Taq DNA polymerase (5 U; Ampliqon, Denmark), 2 μl of bacterial DNA, and 10.5 μl sterile distilled water. Then, the mixtures were incubated with the following conditions in a thermal gradient cycler (Eppendorf, Germany): denaturation at 95°C for 5 min, 35 cycles with denaturation at 95°C for 2 min, annealing at 60°C for 45 s, extension at 70°C for 45 s and final extension at 72°C for 10 min. Finally, PCR products were run with 1% agarose gel in Tris/Borate/EDTA for 40 min and the gel documentation system was used for visualizing them on the gel.

### 2.5. Screening of biofilm formation in isolates

Most importantly, the biofilm formation ability among confirmed *S. aureus* and *P. aeruginosa* isolates was tested using the microtiter plate method as described before with some modifications (Mirzaei et al., 2022b). In the first step, fresh colonies were grown in 5 ml TSB containing 1% glucose with 180 rpm shaking at 37°C overnight, and then the OD of bacteria was set on 0.5 McFarland and then 100 μl diluted suspension containing 10 ^7^ colony-forming units (CFUs) was added to 900 μl of TSB containing 1% glucose, and finally, 200 μl of this suspension containing 2 × 10 ^6^ CFUs was added to the wells of 96 U-shape microplate and afterward overnight incubated at 37°C with shaking at 60 rpm. Afterward, the contents of the wells were outed, and wells were washed with normal saline and air-dried. Finally, 200 μl of 100% methanol was injected into wells and aspirated after 15 min, and then air-dried at room temperature again and, in the next step, were stained with 0.05% crystal violet at a volume of 200 μl for 5 min, and after which the stain was outed. The wells were washed with normal saline and air-dried again. Eventually, 200 μl of 100% ethanol was entered into wells and mixed for 30 min at 37°C while shaking, and then absorbance of wells at 595 nm after transferring their contents to the new wells in the new microplate was recorded *via* a microplate reader (BioTek, United States). TSB with 1% glucose devoid of bacteria served as the negative control, while *S. aureus* ATCC 29213 and *P. aeruginosa* PAO1 served as the positive controls. Briefly, a cut-off OD (ODc) was defined as three standard deviations (SD) above the mean OD of the negative control (uninoculated medium): ODc = average OD of negative control + (3 × SD of negative control). The biofilm production capability of the tested isolates was categorized as follows: OD ≤ OD cut-off (ODc), non-biofilm forming; ODc < OD ≤ 2 × ODc, weak biofilm-forming; 2 × ODc < OD ≤ 4 × ODc, moderate biofilm-forming; and 4 × ODc < OD, strong biofilm-forming (Mirzaei et al., 2022c). Finally, in this study, 20 biofilm-producer strains of MRSA and *P. aeruginosa* were selected including clinical isolates, PAO1, and ATCC for further evaluation.

### 2.6. Antibiotic susceptibility pattern and MDR isolates

Finally, for the determination of antibiotic susceptibility and MDR patterns of MRSA isolates, the Kirby-Bauer procedure was done according to CLSI recommendations as above mentioned for the following antibiotics: clindamycin (CD; 2 μg), trimethoprim-sulfamethoxazole (TS; 1.25 μg), gentamicin (GM; 10 μg), erythromycin (E; 15 μg), and linezolid (LZD; 30 μg) for *S. aureus*, as well as nalidixic acid (NA; 30 μg), colistin (CT; 10 μg), ampicillin (AMP; 10 μg), piperacillin (PRL; 100 μg), imipenem (IMP; 10 μg), cefepime (CPE; 30 μg), and chloramphenicol (C; 30 μg) for *P. aeruginosa* (Wayne, 2010). *S. aureus* ATCC 25923 was applied as the quality control. Finally, by observing at least one or more antibiotic resistances for three or more classes of antibiotics, MDR in selected MRSA and *P. aeruginosa* isolates was characterized (Mirzaei et al., 2022a).

### 2.7. Minimum inhibitory concentration (MIC), minimum bactericidal concentration (MBC), and MBC/MIC

The MIC values for melittin, gentamicin, vancomycin, ciprofloxacin, and rifampin were determined *via* microdilution assay with some changes according to CLSI guidelines for selective isolates (Wayne, 2010; Mirzaei et al., 2022a). In the first step, the fresh bacterial colonies were cultured overnight in MHB with 180 rpm shaking at 37°C, and then, the OD of bacteria was set to 0.5 McFarland as above-mentioned and quickly reached 10 ^6^ CFUs in MHB. In addition, 100 μl of antimicrobial agent serial dilutions were simultaneously made on MHB in 96-well F-bottom microplates. The concentrations of melittin, gentamicin, ciprofloxacin, vancomycin, and rifampin were between 0.156–10, 0.25–512, 0.125–128, 0.5–64, and 0.007–64 μg/ml, respectively. Eventually, 100 μl of the provided suspension equal to 10 ^5^ CFUs was added to wells of the serially diluted antimicrobial agents, and the microplate was overnight incubated at 37°C and then, the lowest concentration of the tested antimicrobial agents caused full inhibition of observable growth was considered as MIC.

The MBC values of melittin, gentamicin, ciprofloxacin, rifampin, and vancomycin were determined using broth microdilution assay with some modifications per CLSI guidelines (Wayne, 2010; Mirzaei et al., 2022a). In brief, the bacterial colonies were cultured overnight in the MHB at 37°C with 180 rpm shaking, and then, the number of bacteria was set to the 0.5 McFarland as above-mentioned and then reached 10 ^6^ CFUs in MHB. In addition, as mentioned above, 100 μl of serial dilutions of antimicrobial agents were simultaneously made on MHB in 96-well F-bottom microplates. Eventually, 100 μl of the suspension equal to 10 ^5^ CFUs was added to wells of the serially diluted antimicrobial agents, and the microplates were overnight incubated at 37°C, and then, 10 μl was cultured on the MHA overnight and grew colonies were determined. Finally, the MBC for melittin, gentamicin, vancomycin, ciprofloxacin, and rifampin was defined as the minimum concentration necessary to kill 100% of cultured bacteria (Wayne, 2010; Mirzaei et al., 2022a). For MIC and MBC of antibiotics, we tested gentamicin and ciprofloxacin for *P. aeruginosa* and vancomycin and rifampin for *S. aureus*, respectively. Additionally, the MBC/MIC values were determined to identify the presence or absence of antibiotic tolerance in isolates (Traczewski et al., 2009).

### 2.8. Minimum biofilm inhibitory concentration (MBIC), and minimal biofilm eradication concentration (MBEC) against biofilm

The MBIC of melittin, gentamicin, ciprofloxacin, vancomycin, and rifampin on the 24 h preformed biofilm was investigated. Regarding this, fresh bacterial colonies were grown overnight in 5 ml of TSB with 1% glucose at 37°C with 180 rpm shaking. Afterward, the OD of cells was set to 0.5 McFarland, as stated previously. Then an inoculation of 2 × 10 ^6^ CFUs provided as described above in TSB with 1% glucose was entered into wells of U-shape microplate and incubated at 37°C overnight with 60 rpm shaking. On the next day, the content of the wells of the U-shape microplate was carefully disposed of and rinsed with normal saline. Simultaneously, melittin at a range of 20 to 0.625 μg/ml and antibiotics at a range of 256 to 1 μg/ml were serially diluted in normal saline at the volume of 100 μl and applied to a microplate and then incubated overnight at 37°C and the amount of biofilm was then quantified as above-mentioned. The MBIC was defined as the minimum concentration of tested agents that inhibited biofilm formation by 90%. The percentage of inhibition of biofilm was computed as follows (Bardbari et al., 2018): MBIC = [1-(OD test/OD control)] × 100.

The MBEC experiment was conducted using the same 96 U-shape microplate as MBIC, to evaluate the biofilm degradative, as well as the killing potential of embedded bacterial in biofilm for melittin, gentamicin, ciprofloxacin, vancomycin, as well as rifampin (Mirzaei et al., 2022b). Briefly, the biofilm of isolates was allowed to produce, as mentioned above, in TSB with 1% glucose. The wells’ contents were then disposed of and rinsed with normal saline. Simultaneously, 100 μl of serially diluted melittin in a range of 20 to 1.25 μg/ml and antibiotics at a range of 1,024 to 2 μg/ml in 100 μl of normal saline were added to the wells. The microplates were incubated overnight at 37°C. On the next day, the contents of the wells were outed, the wells were washed with normal saline, and 100 μl of fresh normal saline was added to the wells and mixed. Then 10 μl of this content was cultured on MHA at 37°C for 48 h, and finally, the number of grown bacterial colonies was determined. The MBEC was considered as the minimum quantity of tested agents to 100% killing of the biofilm-embedded bacteria. It should be noted that we tested gentamicin, and ciprofloxacin for *P. aeruginosa* and also vancomycin and rifampin for *S. aureus*, respectively.

### 2.9. Synergistic effects of melittin and antibiotics toward biofilm

The anti-biofilm effect of melittin, gentamicin, ciprofloxacin, vancomycin, and rifampin in combination was surveyed *via* the microdilution method with some modifications (Mirzaei et al., 2022b). In this regard, fractional antibiofilm indices for MBIC named fractional biofilm inhibitory concentration index (FBICi) of antibiofilm agents against selected biofilm-forming MDR-MRSA and MDR-*P. aeruginosa* isolates were calculated. In brief, at first, 24 h preformed biofilm was generated in 96 U-shape microplates as mentioned above, and then serial dilutions of melittin at a range of 20 to 0.625 μg/ml and antibiotics at a range of 256 to 1 μg/ml at a volume of 100 μl were added to each well and microplate was incubated overnight at 37°C. Finally, the FBICi for combined agents were determined as follows: (MBIC agent 1 in combination/ MBIC agent 1 alone) + (MBIC agent 2 in combination/MBIC agent 2 alone). FBICi refers to the interaction based on the theses findings: Synergy if the conclusion was ≤0.5; Partial synergy if the discovery was 0.5 < to <1; Additive if the result was equal to1; Indifferent if the finding was 1 < to <4; Antagonistic if the finding was 4 ≤ (Mirzaei et al., 2022b). It should be noted that gentamicin, ciprofloxacin for *P. aeruginosa*, vancomycin, and rifampin for *S. aureus* were tested, respectively.

### 2.10. Effect of melittin on the biofilm encoding genes

Selected biofilm-forming strains similar to synergism testing were chosen further for real-time PCR analysis to survey the expression of biofilm-encoding genes *icaA* in *S. aureus* and *LasR* in *P. aeruginosa*. Selected strains were exposed to sub-MIC concentrations of melittin in the range of 5 to 0.039 μg overnight, and on the next day, total RNA was extracted by the extraction kit based on the manufacturer’s recommendations (Gene All, South Korea). The concentration, purity, and integrity of the extracted RNAs were assessed. Then, 1 μg of RNA was then utilized for cDNA synthesis *via* RT-PCR kit according to the manufacturer’s instructions. In the next step, using the 2X Q-PCR Master Mix with 2 μl of cDNA and 1 μl of each *icaA*, *LasR,* and *16S rRNA* primers in a volume of 20 μl on the real-time-PCR equipment (LightCycler^®^ 96 Instrument, Roche, United States), gene expression was measured. The *icaA*, *LasR*, and *16S rRNA* primers were obtained from previous works (Atshan et al., 2012; Koohsari et al., 2016; Abdelraheem et al., 2020; Bai et al., 2020). Initial denaturation took place at 95°C for 10 min, and then 40 cycles of 95°C for 15 s, annealing at 60°C for 45 s, and extension at 72°C for 30 s. To control the amplification efficiency, the standard curve was designed using the serial dilution of mRNA of untreated ATCC 29213 for the *icaA*, and untreated *P. aeruginosa* PAO1 for the *LasR*. Finally, gene expression was calculated *via* Ct assay, and *16S rRNA* genes were used as the internal controls for each bacterium (Rao et al., 2013).

### 2.11. Toxicity assays

The host cell cytotoxicity of melittin was evaluated by the MTT test, as described before (Akbari et al., 2019). Briefly, the HEK-293 cell line was grown in DMEM (containing 10% FCS and antibiotics (100 U/ml penicillin and 100 U/ml streptomycin)). In the next step, the cells were incubated with 5% CO2 and relative humidity equal to 95% at 37°C, and they reached 4 × 10 ^4^ per well and cultured overnight. After 24 h, the serial dilutions of melittin in the range of 5 to 0.039 μg were applied to the wells of a 96-well microplate and incubated for 24 h at 37°C, and afterward, 20 ml of MTT reagent with the concentration of 5 mg/ml was applied to each well, followed by a 4 h incubation. Finally, the supernatant was outed, and 100 ml of DMSO was added to the wells. Absorbance was ultimately determined at 570 nm *via* the spectrophotometer reader (BioTek, United States). The proportion of surviving cells was computed as follows: Percent of survival = (OD test/OD control) × 100 (Akbari et al., 2019).

Besides, to survey the hemolytic effect, several melittin concentrations were utilized per the previously reported approach (Zarrinnahad et al., 2018). A healthy volunteer’s heparinized blood was collected, then centrifuged at 3500 rpm for 10 min, and washed three times with PBS; the supernatant was outed, and 100 μl of 2% human red blood cells (RBCs) stock 2% provided in PBS was then entered into each well of a microplate and treated with melittin (from 5 to 0.039 to 5 μg) and incubated at 37°C for 2 h and then centrifuged at 3000 rpm for 10 min, and the OD of released hemoglobin was read at 540 nm using the microplate spectrophotometer reader (BioTek, United States). Besides, for positive control, 200 μl containing 100 μl 2% RBC and 100 μl of 1% Triton X-100, and for the negative control, 200 μl containing 100 μl 2% RBC and 100 μl PBS were used, respectively. Finally, the percentage of RBC hemolysis was determined as follows: [(OD test − OD negative control)/(OD positive control − OD negative control)] × 100 (Zarrinnahad et al., 2018).

### 2.12. Statistical analysis

In all assays, the GraphPad Prism 9 software (GraphPad Software, Inc., La Jolla, CA, United States) was applied for the various statistical techniques. In this sense, a t-test was applied to evaluate the significance of the findings from concentrations of the anti-biofilm effect of melittin and antibiotics in combination. In addition, the ANOVA test was used to compare the survival rate of the HEK-293 cell line exposed to concentrations of melittin and the control, as well as in gene expression between the treated isolated and the control, and also between the FBIC values. It should be noted that findings were reported as the mean ± standard deviation except for the cases stated otherwise and assays were accomplished with a confidence level of 95%, and a value of *p* < 0.05 was considered significant. To describe the correlation between the examined concentrations and the percent of activities, the non-linear regression test was performed. All experiments were done three times.

## 3. Results

### 3.1. Isolates, MRSA, and biofilm production assay

The result of antibacterial susceptibility testing and molecular test of isolates toward FOX disc and *mecA* gene showed that 66.6% (*n* = 20) of *S. aureus* isolates were demonstrated as MRSA. Notably, 75% (*n* = 6), 72% (*n* = 8), 75% (*n* = 3), and 50% (*n* = 3), of the wound, blood, sputum, and urine were methicillin-resistant, respectively. In this regard, there was no correlation between the source and MRSA isolates (*p* = 0.66). Finally, most *S. aureus* and *P. aeruginosa* isolates could produce varying rates of biofilm. In this regard, the minimum and maximum OD for all isolates were 0.1 and 2.9, respectively. Besides, according to these findings, the biofilm production ability of the isolates was categorized as strong, intermediated, and weak producers, as depicted in Table 1. Finally, after entry and exit criteria for determining pathogenic isolates and biofilm formation ability, 9 clinical MRSA and 9 clinical *P. aeruginosa* isolates were chosen and used for further analysis along *S. aureus* ATCC 29213 and *P. aeruginosa* PAO1.

**Table 1:** Findings of antimicrobial susceptibility testing and biofilm of *Staphylococcus aureus* and *Pseudomonas aeruginosa*.

Strain	FOX	E	TS	CD	GM	LZD	NA	CT	PRL	AMP	IPM	CPE	C	MDR/NonMDR	Biofilm producer
ATCC 29213	S	S	S	S	S	S	–	–	–	–	–	–	–	NonMDR	Intermediate
MRSA 1	R	R	S	R	S	S	–	–	–	–	–	–	–	MDR	Intermediate
MRSA 2	R	S	R	S	R	S	–	–	–	–	–	–	–	NonMDR	Strong
MRSA 3	R	R	S	R	R	S	–	–	–	–	–	–	–	MDR	Weak
MESA 4	R	S	S	S	S	S	–	–	–	–	–	–	–	NonMDR	Intermediate
MRSA 5	R	R	R	R	S	S	–	–	–	–	–	–	–	MDR	Strong
MRSA 6	R	R	S	R	R	S	–	–	–	–	–	–	–	MDR	Intermediate
MRSA 7	R	R	R	S	R	S	–	–	–	–	–	–	–	MDR	Weak
MRSA 8	R	S	S	S	S	S	–	–	–	–	–	–	–	NonMDR	Intermediate
MRSA 9	R	R	S	R	R	S	–	–	–	–	–	–	–	MDR	Strong
PAO1	–	–	–	–	–	–	S	S	R	R	S	S	S	NonMDR	Strong
*P. aeruginosa* 1	–	–	–	–	–	–	R	S	R	R	S	R	S	MDR	Intermediate
*P. aeruginosa* 2	–	–	–	–	–	–	S	S	S	S	S	S	S	NonMDR	Weak
*P. aeruginosa* 3	–	–	–	–	–	–	R	S	R	R	S	R	R	MDR	Intermediate
*P. aeruginosa* 4	–	–	–	–	–	–	R	R	R	R	S	S	R	MDR	Strong
*P. aeruginosa* 5	–	–	–	–	–	–	R	S	R	R	S	R	S	MDR	Weak
*P. aeruginosa* 6	–	–	–	–	–	–	R	S	S	S	S	R	R	MDR	Intermediate
*P. aeruginosa* 7	–	–	–	–	–	–	S	S	S	R	S	S	S	Non MDR	Strong
*P. aeruginosa* 8	–	–	–	–	–	–	R	S	R	R	S	R	R	MDR	Intermediate
*P. aeruginosa* 9	–	–	–	–	–	–	R	S	R	R	S	S	R	MDR	Intermediate

Abbreviations: ATCC, American Type Culture Collection; MRSA, methicillin-resistant *S. aureus*; *P. aeruginosa*, *Pseudomonas aeruginosa*; R, resistant; I, intermediate; S, sensitive; FOX, cefoxitin; E, Erythromycin; TS, Trimethoprim-Sulfamethoxazole; CD, Clindamycin; GM, Gentamicin; LZD, Linezolid; NA, nalidixic acid; CT, Colistin; PRL, Piperacillin; AMP, Ampicillin; IPM, Imipenem; CPE, Cefepime; C, Chloramphenicol; MDR, multidrug-resistant.

### 3.2. Antibacterial susceptibility testing and MDR isolates

According to the disk diffusion data for selected clinical isolates, the antibiotic resistance rate of *S. aureus* toward E, TS, CD, GM, and LZD was 60, 30, 50, 50, and 0%, respectively. Additionally, based on disk diffusion data for selected clinical *P. aeruginosa* isolates, the antibiotic resistance rate toward NA, CT, PRL, AMP, IMP, CPE, and C was 70, 10, 70, 80, 0, 50, and 0%, respectively. In total, 60% of MRSA isolates and 70% of *P. aeruginosa* isolates were MDR. In this regard, there was no correlation between the source and MDR isolates (*p* = 0.23). Further detail on antimicrobial susceptibility testing of antibiotics against isolates is depicted in Table 1.

### 3.3. MIC, MBC, and MBC/MIC values

The results showed that melittin suppressed the growth of MRSA and *P. aeruginosa* isolates, with MIC ranging from 0.625 to 2.5 μg/ml for MRSA, and 1.25 to 10 μg/ml for *P. aeruginosa.* The findings also demonstrated melittin’s bactericidal effect on tested isolates, with MBC ranging from 1.25 to 5 μg/ml for MRSA and 1.25 to 10 μg/ml for *P. aeruginosa*, respectively. The value of the geometric mean of MIC for melittin, gentamicin, ciprofloxacin, vancomycin, and rifampin was 2.1, 8.5, 6.4, 3.03, and 0.46 μg/ml, respectively. Besides, the value of the geometric mean of MBC for melittin, gentamicin, ciprofloxacin, vancomycin, and rifampin was 3.18, 103.96, 12.99, 6.06, and 6.4 μg/ml, respectively. Finally, the geometric mean value for the MBC/MIC for melittin, gentamicin, ciprofloxacin, vancomycin, and rifampin was 1.51, 12.12, 2, 2, and 13.92, respectively. Details on MIC and MBC findings are depicted in Table 2.

**Table 2:** MIC, MBC, and MBC/MIC values for melittin, vancomycin, rifampin, gentamicin, and ciprofloxacin against *S. aureus* and *P. aeruginosa.*

Strain	MEL-MIC	MEL-MBC	VAN-MIC	VAN-MBC	RIF-MIC	RIF-MBC	GEN-MIC	GEN-MBC	CIP-MIC	CIP-MBC	VAN-MBC/MIC ratio	RIF-MBC/MIC ratio	GEN-MBC/MIC ratio	CIP-MBC/MIC ratio	MEL-MBC/MIC ratio
ATCC 29213	1.25	5	1	2	0.015	0.5	–	–	–	–	2	32	–	–	4
MRSA 1	2.5	5	4	8	0.5	8	–	–	–	–	2	16	–	–	2
MRSA 2	1.25	2.5	8	16	1	16	–	–	–	–	2	16	–	–	2
MRSA 3	1.25	1.25	2	4	1	8	–	–	–	–	2	8	–	–	1
MESA 4	0.625	1.25	4	8	0.5	4	–	–	–	–	2	8	–	–	2
MRSA 5	2.5	5	8	16	2	16	–	–	–	–	2	8	–	–	2
MRSA 6	1.25	2.5	4	8	1	8	–	–	–	–	2	8	–	–	2
MRSA 7	0.625	1.25	2	4	0.25	4	–	–	–	–	2	16	–	–	2
MRSA 8	2.5	5	4	8	0.25	8	–	–	–	–	2	32	–	–	2
MRSA 9	1.25	2.5	1	2	1	16	–	–	–	–	2	16	–	–	2
PAO1	5	10	–	–	–	–	0.5	8	0.25	1	–	–	16	4	2
*P. aeruginosa* 1	2.5	5	–	–	–	–	8	128	8	16	–	–	16	2	2
*P. aeruginosa* 2	5	5	–	–	–	–	4	64	2	8	–	–	16	4	1
*P. aeruginosa* 3	2.5	5	–	–	–	–	32	256	16	32	–	–	8	2	2
*P. aeruginosa* 4	10	10	–	–	–	–	64	512	32	32	–	–	8	1	1
*P. aeruginosa* 5	2.5	2.5	–	–	–	–	32	512	1	2	–	–	8	2	1
*P. aeruginosa* 6	1.25	2.5	–	–	–	–	16	128	2	4	–	–	8	2	2
*P. aeruginosa* 7	2.5	5	–	–	–	–	4	64	32	64	–	–	16	2	2
*P. aeruginosa* 8	5	10	–	–	–	–	16	128	16	32	–	–	16	2	2
*P. aeruginosa* 9	1.25	1.25	–	–	–	–	2	32	64	64	–	–	16	1	1

Abbreviations: ATCC, American Type Culture Collection; VAN, vancomycin; GEN, gentamicin; CIP, ciprofloxacin; MIC, minimum inhibitory concentration; MBC, minimum bactericidal concentrations; RIF, rifampin, Mel; melittin; MRSA, methicillin-resistant Staphylococcus aureus.

### 3.4. MBIC and MBEC

Most importantly, the findings also demonstrated that melittin suppressed the preformed biofilm of tested isolates, with MBIC values from 10 to 2.5 μg/ml for MRSA and 20 to 5 μg/ml for *P. aeruginosa,* respectively. Besides, the MBIC results for gentamicin, ciprofloxacin, vancomycin, and rifampin ranged from 4 to 128, 2 to 128, 16 to 128, and 8 to 128 μg/ml, respectively. The value of the geometric mean of MBIC for melittin, gentamicin, ciprofloxacin, vancomycin, and rifampin was 7.07, 34.29, 27.85, 39.39, and 25.99 μg/ml, respectively. Besides, the findings also found that melittin eradicated the biofilm-embedded bacteria, with MBEC values ranging from 10 to 40 μg/ml for MRSA and *P. aeruginosa*. Besides, the MBEC ranges for gentamicin, ciprofloxacin, vancomycin, and rifampin were 64 to 512, 8 to 1,024, 64 to 512, and 32 to 1,024/mL, respectively. The value of the geometric mean of the MBEC value for melittin, gentamicin, ciprofloxacin, vancomycin, and rifampin was 20, 207.93, 181.01, 194.01, and 256 μg/ml, respectively. Further details on MBIC and MBEC are shown in Table 3.

**Table 3:** MBIC and MBEC values of melittin, vancomycin, rifampin, gentamicin, and ciprofloxacin against *S. aureus* and *P. aeruginosa*.

Isolate (*n* = 20)	MEL-MBIC (μg/ml)	MEL-MBEC (μg/ml)	Van-MBIC (μg/ml)	Van-MBEC (μg/ml)	Rif-MBIC (μg/ml)	Rif-MBEC (μg/ml)	GEN-MBIC (μg/ml)	GEN-MBEC (μg/ml)	CIP-MBIC (μg/ml)	CIP-MBEC (μg/ml)
*S. aureus* ATCC 29213	10	40	32	128	8	128	–	–	–	–
MRSA 1	5	10	64	256	8	128	–	–	–	–
MRSA 2	10	20	16	64	32	128	–	–	–	–
MRSA 3	5	10	32	128	8	32	–	–	–	–
MESA 4	5	20	16	128	64	512	–	–	–	–
MRSA 5	10	20	64	256	16	512	–	–	–	–
MRSA 6	5	20	128	512	32	256	–	–	–	–
MRSA 7	2.5	20	32	256	64	512	–	–	–	–
MRSA 8	5	20	64	512	32	512	–	–	–	–
MRSA 9	10	40	32	128	128	1,024	–	–	–	–
*P. aeruginosa* PAO1	20	40	–	–	–	–	4	64	2	8
*P. aeruginosa* 1	10	20	–	–	–	–	32	128	8	64
*P. aeruginosa* 2	5	20	–	–	–	–	16	128	32	128
*P. aeruginosa* 3	5	10	–	–	–	–	64	256	16	64
*P. aeruginosa* 4	10	20	–	–	–	–	128	512	64	256
*P. aeruginosa* 5	5	10	–	–	–	–	64	256	64	512
*P. aeruginosa* 6	10	20	–	–	–	–	64	512	16	128
*P. aeruginosa* 7	10	40	–	–	–	–	32	128	128	1,024
*P. aeruginosa* 8	10	40	–	–	–	–	64	256	64	1,024
*P. aeruginosa* 9	5	10	–	–	–	–	16	256	64	512

Abbreviations: MBIC, Minimum biofilm inhibition concentration; MBEC, Minimum biofilm eradication concentration; MRSA, methicillin-resistant *S. aureus*.

### 3.5. Synergistic activity of antimicrobial agents on biofilm

In the current study, the value of the geometric mean for best synergistic melittin–vancomycin concentrations based on FBICi against MRSA 5, MRSA 6, and ATCC 29213 was 0.09, 0.09, and 0.25, respectively. Besides, the value of the geometric mean for best synergistic melittin–rifampin concentrations based on FBICi against MRSA 2, MRSA 9, and ATCC 29213 was 0.35, 0.35, and 0.62, respectively. Besides, the geometric means for best synergistic melittin–gentamicin concentrations based on FBICi against *P. aeruginosa* PAO1, *P. aeruginosa* 4, and *P. aeruginosa* 8 were 0.18, 0.08, and 0.06, respectively. Besides, for best synergistic melittin–ciprofloxacin concentrations based on FBICi against *P. aeruginosa,* PAO1, *P. aeruginosa* 7, and *P. aeruginosa* 8 were 0.37, 0.24, and 0.5, respectively. Further details on antibiofilm synergistic effects of melittin and antibiotics are shown in Tables 4, 5.

**Table 4:** The best synergistic concentrations of vancomycin-melittin and rifampin-melittin against biofilm of selected *S. aureus* isolates.

ATCC 29213		MRSA 5		MRSA 6		ATCC 29213		MRSA 2		MRSA 9	
VAN+MEL (μg/ml)	FBIC indices	VAN+MEL (μg/ml)	FBIC indices	VAN+MEL (μg/ml)	FBIC indices	RIF + MEL (μg/ml)	FBIC indices	RIF + MEL (μg/ml)	FBIC indices	RIF + MEL (μg/ml)	FBIC indices
32 + 10	2	64 + 2.5	1.25	64 + 1.25	1.12	8 + 2.5	1.25	32 + 10	2	128 + 10	2
16 + 5	1.5	32 + 1.25	0.62	32 + 0.625	0.56	4 + 1.25	0.62	16 + 5	1	64 + 5	1
8 + 2.5	0.75	16 + 0.625	0.31	16 + 0.312	0.25	–	–	8 + 2.5	0.5	32 + 2.5	0.5
4 + 1.25	0.37	8 + 0.312	0.15	8 + 0.156	0.14	–	–	4 + 1.25	0.25	16 + 1.25	0.25
2 + 0.625	0.18	4 + 0.156	0.07	4 + 0.078	0.07	–	–	–	–	–	–
–	–	2 + 0.078	0.03	2 + 0.039	0.03	–	–	–	–	–	–

Abbreviations: ATCC, American type culture collection; VAN, vancomycin; MEL; melittin; RIF, Rifampin; MRSA, methicillin-resistant *S. aureus*; FBIC, fractional biofilm inhibitory concentration.

**Table 5:** The best synergistic concentrations of gentamicin-melittin and ciprofloxacin-melittin against biofilm of selected *P. aeruginosa* isolates.

*P. aeruginosa* PAO1		*P. aeruginosa* 4		*P. aeruginosa* 8		*P. aeruginosa* PAO1		*P. aeruginosa* 7		*P. aeruginosa* 8	
GEN + MEL (μg/ml)	FBIC indices	GEN + MEL (μg/ml)	FBIC indices	GEN + MEL (μg/ml)	FIC indices	CIP + MEL (μg/ml)	FBIC indices	CIP + MEL (μg/ml)	FBIC indices	CIP + MEL (μg/ml)	FBIC indices
4 + 10	1.5	64 + 5	1	32 + 2.5	0.75	2 + 10	1.5	128 + 10	2	64 + 10	2
2 + 5	0.75	32 + 2.5	0.5	16 + 1.25	0.37	1 + 5	0.75	64 + 5	1	32 + 5	1
1 + 2.5	0.37	16 + 1.25	0.25	8 + 0.625	0.18	0.5 + 2.5	0.37	32 + 2.5	0.5	16 + 2.5	0.5
0.5 + 1.25	0.18	8 + 0.625	0.12	4 + 0.312	0.09	–	–	16 + 1.25	0.25	–	–
0.25 + 0.625	0.09	4 + 0.312	0.06	2 + 0.156	0.04	–	–	8 + 0.625	0.12	–	–
–	–	2 + 0.156	0.03	1 + 0.078	0.02	–	–	–	–	–	–
–	–	1 + 0.078	0.01	0.5 + 0.039	0.01	–	–	–	–	–	–

Abbreviation: *P. aeruginosa*, *Pseudomonas aeruginosa*; GEN, Gentamicin; MEL, melittin; CIP, Ciprofloxacin; FBIC, fractional biofilm inhibitory concentration.

### 3.6. Activity of melittin on biofilm encoding genes

**Figure 1:**
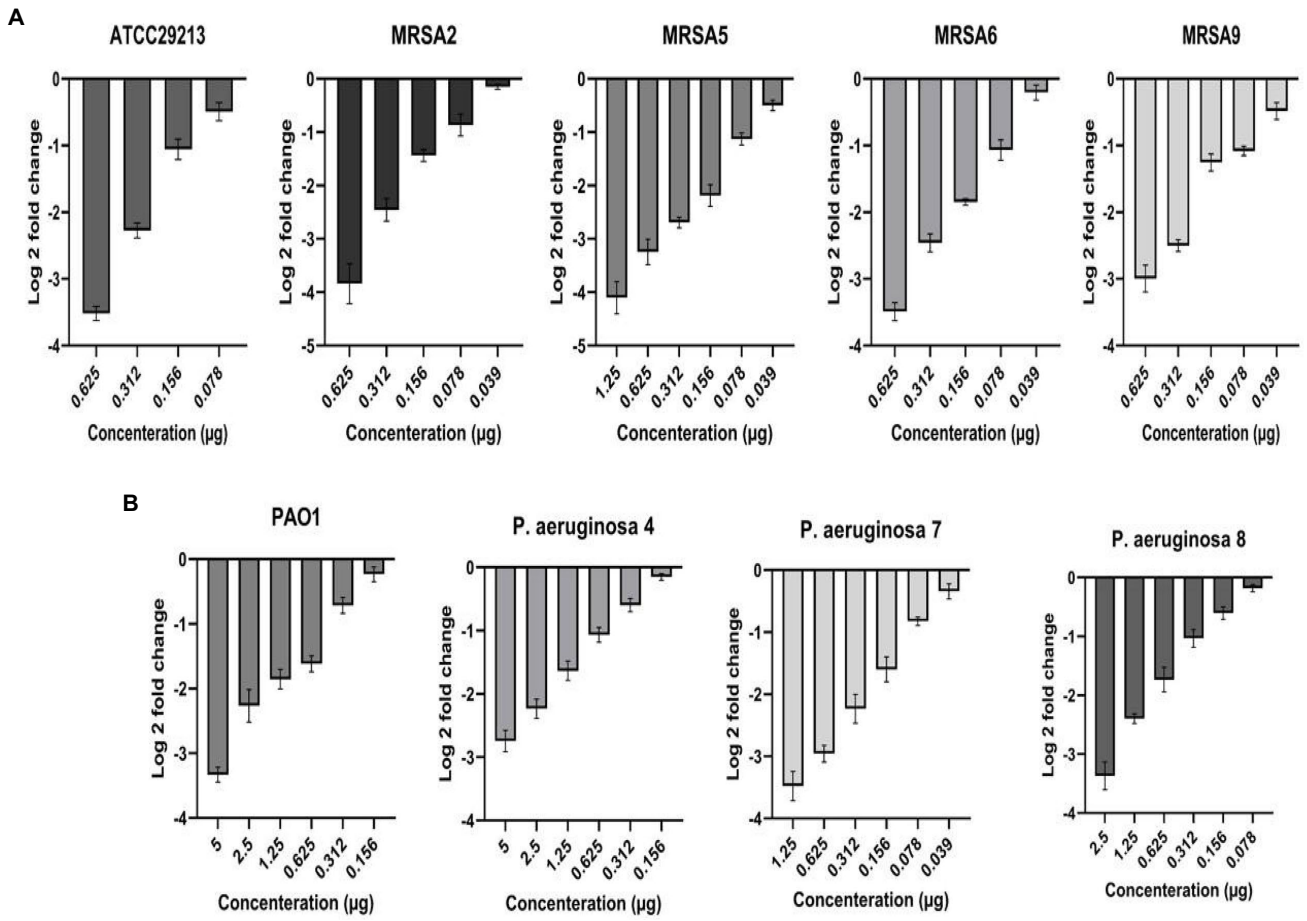
Downregulation of biofilm-associated genes *icaA* **(A)** and *Las* R **(B)** in *Staphylococcus aureus* and *Pseudomonas aeruginosa* at sub-inhibitory concentrations of melittin, respectively.

The activity of sub-lethal melittin concentrations from 5 to 0.039 μg on the expression of the *icaA* and *LasR* was tested for the selected isolates after 24 h. In this regard, log 2-fold change demonstrated that expression of the *icaA* in *S. aureus* and *LasR* in *P. aeruginosa* exposed to sub-MIC melittin were downregulated at a range from 4.11 to 0.1 fold for *icaA*, and 3.7 to 0.11 fold for *LasR*, respectively (Figure 1).

In particular, for *icaA* of *S. aureus* ATCC 29213, at 0.078 to 0.625 μg, the downregulation range was from 0.34 to 3.63. The downregulation range for MRSA 2 at 0.039 to 0.625 μg was from 0.11 to 4.11. The downregulation range for MRSA 5 at 0.039 to 1.25 μg was from 0.4 to 4.4. Besides, the downregulation range for MRSA 6 at 0.039 to 0.625 μg was from 0.1 to 3.6. Finally, the downregulation range for MRSA 9 at 0.039 to 0.625 μg was from 0.35 to 3.2. On the other side, for *LasR* of *P. aeruginosa* PAO1, at 0.156 to 5 μg, the downregulation range was from 0.1 to 3.4. The downregulation range for *P. aeruginosa* 4 at 0.156 to 5 μg was from 0.1 to 2.93. The downregulation range for *P. aeruginosa* 7 at 0.039 to 1.25 μg was from 0.22 to 3.7. Finally, the downregulation range for *P. aeruginosa* 8 at 0.078 to 2.5 μg was from 0.11 to 3.54. In this sense, a linearity relationship was found at evaluated concentrations of melittin, and it also indicated that the downregulation of biofilm-associated genes was generally dose-dependent (R^2^ = 0.89). Besides, ANOVA showed a statistical difference in biofilm-associated genes between the treated and untreated samples (*p* < 0.05).

### 3.7. Cytotoxicity and hemolytic activity of melittin

The cytotoxicity findings ranged from 5, 2.5, 1.25, 0.625, 0.312, and 0.156 μg of melittin 85.9, 69.4, 45.3, 25.2, 10, and 3.5% cytotoxicity on HEK-293 was seen, respectively. Notably, at the best synergistic concentrations of melittin with antibiotics, i.e., 0.078 and 0.039 μg, this peptide did not show any toxicity toward the HEK-293 (Table 6). Of note, a t-test demonstrated no difference between the survival rate of 0.078 and 0.039 μg of melittin and the control sample (*p* = 0.085). Finally, the hemolytic effect of melittin against RBCs ranging from 5, 2.5, 1.25, 0.625, 0.312, and 0.156 μg was 91.6, 80.5, 74.2, 59.5, 25, and 6%, respectively, whilst melittin with 0.078 and 0.039 concentrations showed 0% hemolysis on RBCs (Table 6).

**Table 6:** Overview of toxicity and hemolysis of melittin at the best synergistic concentrations.

Melittin (μg/ml)	Cell death (%)	RBC hemolysis (%)
5	85.9 ± 3.4	91.6 ± 2.4
2.5	69.4 ± 3.2	80.5 ± 2
1.25	45.3 ± 3.5	74.2 ± 1.8
0.625	25.2 ± 3.1	59.5 ± 2.1
0.312	10 ± 2.1	25 ± 1.7
0.156	3.5 ± 2.2	6 ± 2
0.078	0	0
0.039	0	0

## 4. Discussion

MDR pathogens are difficult to treat with antibiotics, especially when they generate biofilm, and these pathogens are a leading source of death in some cases, such as burn and *CF* patients, as well as infection in diabetes patients with chronic non-healing wounds (Emerson et al., 2002; Hiramatsu et al., 2014; Akbari et al., 2019). There is a critical need for novel antimicrobial agents that more effectively target biofilm and its embedded bacteria because nearly all antimicrobials currently used in clinics are active against planktonic growing bacteria. Regarding this, among the limited number of novel agents under investigation, AMPs have shown to be promising to ensure their advancement as active agents toward MDR bacterial infections, as well as potential targets for novel antibiofilm therapeutics (Hale and Hancock, 2007).

Our findings showed that melittin suppressed the growth of isolates, with MIC from 0.625 to 2.5 μg/ml for MRSA and 1.25 to 10 μg/ml for *P. aeruginosa.* The findings also demonstrated the bactericidal effect of melittin on tested isolates, with MBC from 1.25 to 5 μg/ml for MRSA and 1.25 to 10 μg/ml for *P. aeruginosa*, respectively. A comparison of the antibacterial effects of melittin on MRSA and MDR methicillin-resistant *Staphylococcus epidermidis* (MRSE), MDR *P aeruginosa,* and MDR *Acinetobacter baumannii* by others (Choi et al., 2015a; Akbari et al., 2019; Mirzaei et al., 2022a) shows the same result as our findings. Besides, the value of the geometric mean of MBC of the gentamicin, ciprofloxacin, vancomycin, and rifampin isolates was 8.5, 6.4, 3.03, and 0.46 μg/ml, respectively. The geometric mean value of MBC for gentamicin, ciprofloxacin, vancomycin, and rifampin for all isolates was 103.96, 12.99, 6.06, and 6.4 μg/ml, respectively. It has been found that melittin binds to bacterial membranes and creates pores, resulting in osmotic bacterial lysis (Bevalian et al., 2021).

Most importantly, our investigation of MBIC and MBEC demonstrated the potent anti-biofilm action of melittin toward all tested isolates with MBIC from 2.5 to 10 μg/ml for MRSA and 5 to 20 μg/ml for *P. aeruginosa*, as well as MBEC from 10 to 40 μg/ml for MRSA and *P. aeruginosa,* respectively. These findings are per our previous study on the strong biofilm of MDR MRSE (Mirzaei et al., 2022b) as well as by others on strong biofilm-forming MDR *A. baumannii* strains (Bardbari et al., 2018), biofilm-forming solid MDR *P. aeruginosa* (Khozani et al., 2019), and biofilm of MRSA (Lima et al., 2021). Besides, the MBIC results for gentamicin, ciprofloxacin, vancomycin, and rifampin were in the range 4 to 128, 2 to 128, 16 to 128, and 8 to 128 μg/ml, respectively, and MBEC results for gentamicin, ciprofloxacin, vancomycin, and rifampin were 64 to 512, 8 to 1,024, 64 to 512, and 32 to 1,024 μg/ml, respectively. Our findings are higher than those reported by others such as Douthit and colleagues who noted that MBIC values for vancomycin and rifampin were 1 μg/ml and 80 ng/ml, respectively, and also, MBEC of vancomycin and rifampin was 6 μg/mL and 80 ng/ml, respectively, against biofilm of *S. aureus* (Douthit et al., 2020). Besides, the determined MBEC gentamicin was >1,490 μg against *P. aeruginosa* by Das et al. (2016) is higher than our study.

Our results also found a synergistic effect of melittin in combination with antibiotics toward biofilm-forming MDR-MRSA and MDR-*P. aeruginosa*. The geometric mean values for best synergistic melittin–vancomycin and melittin–rifampin concentrations based on FBICi against *S. aureus* were 0.12 and 0.42, respectively. Besides, the geometric mean values for best synergistic melittin–gentamicin and melittin–ciprofloxacin concentrations based on FBICi against *P. aeruginosa* were 0.09 and 0.3, respectively. Some reports noted melittin for its synergistic effect on biofilm when used with antibiotics. Mohammadi et al. (Bardbari et al., 2018) found that melittin has a highly synergistic impact with imipenem and colistin toward the biofilm of MDR *A. baumannii*. Maiden et al. (2019) found that melittin acted alone and/or in combination with tobramycin to kill biofilm-embedded *P. aeruginosa*. Besides, our previous results found the synergistic action of melittin with rifampin and vancomycin toward potent biofilm of MDR-MRSE (Mirzaei et al., 2022b). These findings are in agreement with our results.

The synergy caused by melittin with antibiotics is most likely related to the site of their action on the bacterial membrane, cell wall, RNA polymerase, DNA gyrase, and protein synthesis inhibition (Zarghami et al., 2021b, 2022; Mirzaei et al., 2022a). Melittin disrupts the integrity of the cell membrane and creates pores that probably facilitate the penetration of antibiotics into the bacteria, and in the next step, the antibiotics inhibit the growth of the bacteria and also kill the bacteria through the mentioned targets (Lee et al., 2013; Światły-Błaszkiewicz and Mrówczyńska, 2020; Zarghami et al., 2021a, 2022; Akbari et al., 2022). Most importantly, melittin has been found to have some convergent anti-biofilm mechanisms, including membrane degradation of biofilm-embedded bacteria, degradation of biofilm matrix, and downregulation of genes responsible for biofilm formation (Bardbari et al., 2018; Khozani et al., 2019; Shams et al., 2020; Zarghami et al., 2021b). For further determination of the anti-biofilm effect of melittin and its underlying mechanism of biofilm attenuation, real-time PCR for biofilm encoding genes was done. Hence, it found that biofilm-associated genes *icaA* in *S. aureus* and *LasR* in *P. aeruginosa were downregulated in all tested isolates at a range from 4.11 to 0.1 fold for icaA* and from 3.7 to 0.11 fold for *LasR*, respectively. These results are in agreement with our previous work (Mirzaei et al., 2022b), that sub-MIC of melittin significantly downregulated *icaA* expression in MDR MRSE, and also Mohammadi et al. (Bardbari et al., 2018), found that sub-MIC of melittin significantly decreased the biofilm-associated *bap* gene expression in MDR *A. baumannii*.

Along with arresting the emergence of antibiotic-resistant bacterial mutants, a further goal of multidrug therapy is to decrease the risk of single-drug toxicity to improve the quality of life for patients. In this regard, it has been found that melittin could disrupt the phospholipids packaging in the lipid bilayer, causing pore formation, resulting in the lysis of human RBC (Światły-Błaszkiewicz and Mrówczyńska, 2020). Our findings found synergistic melittin concentrations to destroy biofilm did not have cytotoxicity or hemolytic activities. However, the hemolytic effect of melittin alone (0.625 and 1.25 μg) on erythrocytes was toxic (25–45% cell death); therefore, we recommend that this AMP be used in combination and also in future studies, the ability to modify the melittin sequence to reduce cytotoxicity and improve bactericidal effects can be investigated. This peptide has shown various antimicrobial effects in preclinical *in vitro* and *in vivo*, and despite convincing efficacy data, its applicability to humans may be met with challenges due to issues including its non-specific cytotoxicity, degradation, and hemolytic activity. Hence, some optimization approaches, including the utilization of melittin-derived peptides, nanoparticle-based delivery of melittin, and combination therapy, can circumvent the issues. More importantly, reducing the concentration of antibiotics in synergism with melittin AMP can decrease drug side effects, especially in patients with kidney failure. Our results are consistent with the cytotoxic and hemolytic effects of melittin performed by others (Akbari et al., 2019; Mirzaei et al., 2022a).

## 5. Conclusion

As the occurrence of MDR pathogens is rising, the need for novel antimicrobials and ways to potentiate conventional antibiotics is crucial. Besides, as most conventional antibiotics are only active against proliferating planktonic bacteria, hence, eradicating persisters embedded in biofilm is difficult, and also, the biofilm matrix acts as a pharmacokinetic barrier, restricting the diffusion of antimicrobial agents and other noxious substances into the biofilm matrix. Accordingly, the use of newer and combination approaches to control and eradicate biofilm-mediated infection is one of the crucial requirements. In this regard, we found that melittin has a good effect against MDR MRSA and MDR *P. aeruginosa* as well as mature biofilms of both pathogens alone and in combination with conventional antibiotics. We also demonstrated the synergistic effects of melittin and antibiotics at low concentrations, suggesting that decreasing the concentration of antimicrobial drugs required for therapy can decrease their cytotoxic effects. Hence, these findings show that melittin can be a promising candidate for further evaluation *in vivo* and clinical biofilm-associated infection by MDR pathogens alone or in combination with antibiotics.

## Data availability statement

The original contributions presented in the study are included in the article/Supplementary materials, further inquiries can be directed to the corresponding author.

## Author contributions

RM performed all experiments and analyses, and also wrote the manuscript. HEGG served as advisor. RR contributed as a supervisor and also in the revision of the manuscript. All authors contributed to the article and approved the submitted version.

## Conflict of interest

The authors declare that the research was conducted in the absence of any commercial or financial relationships that could be construed as a potential conflict of interest.

## Publisher’s note

All claims expressed in this article are solely those of the authors and do not necessarily represent those of their affiliated organizations, or those of the publisher, the editors and the reviewers. Any product that may be evaluated in this article, or claim that may be made by its manufacturer, is not guaranteed or endorsed by the publisher.

## Abbreviations

MDR: Multidrug-resistant
MRSA: methicillin-resistant *S. aureus*
AMPs: antimicrobial peptides
ABPs: antibiofilm peptides
MSA: Mannitol Salt Agar
MHA: Mueller Hinton Agar
MHB: Mueller Hinton broth
TSB: Trypticase soy broth
FBS: Fetal bovine serum
DMEM: Dulbecco’s Modified Eagle’s Medium
FCS: Fetal-Calf Serum
MTT: 3-(4, 5-dimethyl-2-thiazolyl)-2, 5-diphenyl-2 H-tetrazoliumbromide
DMSO: dimethyl sulfoxide
PCR: polymerase chain reaction
OD: optical density
CFUs: colony-forming units
MIC: minimum inhibitory concentration
MBC: minimum bactericidal concentration
MBIC: minimum biofilm inhibitory concentration
MBEC: minimal biofilm eradication concentration
FBICi: fractional biofilm inhibitory concentration index
MRSE: methicillin-resistant *Staphylococcus epidermidis*
